# A Duplex PCR Assay for the Detection of *Ralstonia solanacearum* Phylotype II Strains in *Musa* spp.

**DOI:** 10.1371/journal.pone.0122182

**Published:** 2015-03-26

**Authors:** Gilles Cellier, Aurélie Moreau, Aude Chabirand, Bruno Hostachy, Florent Ailloud, Philippe Prior

**Affiliations:** 1 French Agency for Food, Environmental and Occupational Health & Safety (ANSES), Plant Health Laboratory (LSV), Tropical Pests and Diseases Unit, Saint Pierre 97410, Reunion, France; 2 Centre de coopération internationale en recherche agronomique pour le développement (CIRAD), Unité Mixte de Recherche (UMR) Peuplements Végétaux et Bioagresseurs en Milieu Tropical (PVBMT), Saint Pierre 97410, Reunion, France; 3 Institut national de la Recherche Agronomique (INRA), Department of Plant Health and Environment (SPE)—Centre de coopération internationale en recherche agronomique pour le développement (CIRAD), Unité Mixte de Recherche (UMR) Peuplements Végétaux et Bioagresseurs en Milieu Tropical (PVBMT), Saint Pierre 97410, Reunion, France; Dong-A University, REPUBLIC OF KOREA

## Abstract

Banana wilt outbreaks that are attributable to Moko disease-causing strains of the pathogen *Ralstonia solanacearum* (Rs) remain a social and economic burden for both multinational corporations and subsistence farmers. All known Moko strains belong to the phylotype II lineage, which has been previously recognized for its broad genetic basis. Moko strains are paraphyletic and are distributed among seven related but distinct phylogenetic clusters (sequevars) that are potentially major threats to *Musaceae*, *Solanaceae*, and ornamental crops in many countries. Although clustered within the Moko IIB-4 sequevar, strains of the epidemiologically variant IIB-4NPB do not cause wilt on Cavendish or plantain bananas; instead, they establish a latent infection in the vascular tissues of plantains and demonstrate an expanded host range and high aggressiveness toward *Solanaceae* and *Cucurbitaceae*. Although most molecular diagnostic methods focus on strains that wilt *Solanaceae* (particularly potato), no relevant protocol has been described that universally detects strains of the *Musaceae*-infecting Rs phylotype II. Thus, a duplex PCR assay targeting Moko and IIB-4NPB variant strains was developed, and its performance was assessed using an extensive collection of 111 strains representing the known diversity of Rs Moko-related strains and IIB-4NPB variant strains along with certain related strains and families. The proposed diagnostic protocol demonstrated both high accuracy (inclusivity and exclusivity) and high repeatability, detected targets on either pure culture or spiked plant extracts. Although they did not belong to the Moko clusters described at the time of the study, recently discovered banana-infecting strains from Brazil were also detected. According to our comprehensive evaluation, this duplex PCR assay appears suitable for both research and diagnostic laboratories and provides reliable detection of phylotype II Rs strains that infect *Musaceae*.

## Introduction

The Cavendish banana and plantain (cooking banana) (*Musa* spp.) are among the most economically important crops, and they also represent staple foods in developing countries. However, various pathogens affect their production, and the most epidemiologically active pathogens are *Fusarium oxysporum* f.sp. *cubense* race 4 [1], banana bunchy top virus [2], and two bacteria that cause bacterial wilt, namely *Xanthomonas vasicola* pv. *musacearum* [3] and *Ralstonia solanacearum* (Rs) [4]. Bacterial wilt caused by Rs on both bananas and plantains continues to be a major constraint on the production of these crops, both for multi-national corporations and for subsistence farmers [5].

Recognized as a species complex (Rssc) [6], Rs is phylogenetically classified into four groups, called phylotypes, that take into account the phylogeography and evolutionary histories of the various strains [6,7]. The four groups are Asian phylotype I; American phylotype II, which encompasses the Moko and 4NPB strains; African phylotype III; and Indonesian phylotype IV, which encompasses the closely related species *R*. *syzygii* (Sumatra disease of clove trees) and the blood disease bacterium (BDB) [8,9]. A recently proposed taxonomic revision divides the Rssc into three genomic species [10]. While phylotype II is classified as a separate genomic species, its name remains *R*. *solanacearum*. The Rssc comprises strains that are capable of causing wilt in *Musaceae* plants and that cluster into two distant phylogenetic groups: (i) Moko disease-causing strains reported from Latin America, Asia, and the Philippines (Moko is recognized as Bugtok disease [11,12]) and (ii) the BDB originating in Indonesia and Malaysia. Systemic vascular infection by Rs induces symptoms that begin with the yellowing of leaves and tissue necrosis and that lead to a general collapse of the plant. The fruits are inedible and exhibit internal vascular discoloration. Specific symptoms can be observed, particularly with BDB, which produces a reddish coloration of the vascular ring in the fruit [13]. Bugtok disease only affects the floral bud, leading to hardening of the fruit (stone fruit) [14].

Phylotype II harbors the largest number of epidemiologically active ecotypes, such as Brown rot, Moko, NPB, and Granville wilt. As a working definition, the phylotypes are further subdivided into sequevars [6]. The Moko disease-causing strains are paraphyletic and have historically clustered into four sequevars: IIA-6, IIA-24, IIB-3, and IIB-4. The pathological variant IIB-4NPB was first reported in diseased anthurium (*Anthurium andreanum*) in Martinique [15] and was phylogenetically assigned to the Moko lineage IIB-4. These strains are variants that are not pathogenic to bananas (NPB) but that demonstrate a host range that expands to *Cucurbitaceae*.

The Moko-associated strains, in addition to being soil-borne and transmitted through wounds and cuttings, can also be actively transmitted by insects through the bud [16]; the pathogen then migrates down into the plant, leading to symptoms that start with fruit decay and end in plant collapse. In addition to these two groups, the epidemiological 4NPB lineage variant, which is grouped into the Moko sequevar IIB-4 lineage, does not cause wilt on Cavendish or plantain bananas; instead, this variant establishes itself and moves within the vascular tissues of plantains, even via soil-borne contamination, as it establishes a latent infection through the root system [15].

Recently, unanticipated Moko disease-related strains from Brazil were reported [17] that clustered (i) into the previously described *Solanaceae*-related sequevars IIA-41 and IIB-25, which are not related to historic Moko lineages, and (ii) into a newly proposed sequevar, IIA-53. This finding highlights the fact that the Moko ecotype benefits from a broad genetic basis and harbors far more genetic diversity than anticipated [17,18]. The current Moko strain-specific molecular diagnostic method consists of a *Musa* multiplex PCR (Mmx-PCR) [19,20] that targets the historically known sequevars IIA-6, IIA-24, IIB-3, and IIB-4 by producing a size-specific amplification band for each Moko sequevar. IIB-4 Moko strains also produce another specific amplification band that is not observed with IIB-4NPB strains. However, this protocol was unable to specifically detect the Moko disease-related strains reported in Brazil by Albuquerque et al. [17], as it relies on the characterization of historically known sequevars. Therefore, it appears that there is no official diagnostic method suitable for the specific detection of phylotype II strains that infect bananas. There is a strong need for such a method when conducting territory and border surveillance, for basic material *in vitro*, and for plantlet banana production, as these processes must be free of quarantine-propagative pathogens, including Rssc strains. In addition to representing a major threat to the banana trade and to sustainable production, these strains may also threaten *Solanaceae* production, as most of them are pathogenic to potato (*Solanum tuberosum*) or tomato (*S*. *lycopersicum*) under artificial conditions [21].

Based on the whole-genome sequences of Moko strains, we developed a robust, simple, and affordable duplex PCR assay that is specific for phylotype II Rssc strains that can be retrieved from banana and plantain tissues (Moko disease-causing strains and IIB-4NPB pathological variants). Here, we present an extensive characterization of the performance of the duplex PCR assay.

## Materials and Methods

### Bacterial strains and viruses

A set of 111 reference strains was selected to cover the known genetic diversity among Rssc strains and strains related to the Rssc (Table 1). Within the Rssc, a total of 40 bacterial strains were selected as targets, while 45 bacterial strains were selected as non-targets. Additionally, 21 non-target bacterial strains that are phylogenetically close to the Rssc or related to banana diseases were selected. Finally, 5 viruses were selected as being related to banana diseases. The bacterial strains were obtained from the *Centre de coopération Internationale en Recherche Agronomique pour le Développement* (CIRAD—Saint Pierre, Reunion Island) and were stored at -80°C on cryobeads (Microbank, Pro-labs Diagnostics, Toronto, Canada). The bacteria were cultured overnight in Luria-Bertani broth (LB) at 28°C with 250 rpm agitation, streaked on modified Sequeira semi-selective solid medium containing agar (18 g/L), yeast extract (1 g/L), peptone (11 g/L), glycerol (6.3 g/L), crystal violet (2 mg/L), polymyxin-β-sulfate (10 mg/L), tyrothricine (20 mg/L), chloramphenicol (5 mg/L), 2,3,5-triphenyltetrazolium chloride (11 mg/L), Tilt (Propiconazole; Syngenta, Bâle, Switzerland; 0.004%), and penicillin (20 U) and incubated for 48h at 28°C. Calibrated bacterial suspensions were generated in 0.1 M Tris-HCl (pH 7.1) (Sigma-Aldrich, Saint-Louis, MO, USA) adjusted initially to 10^8^ CFU/mL, as determined by measuring an optical density of 0.1 at 650 nm (Biomate 3, Thermo Scientific, Boston, MA, USA). Successive dilutions were prepared using molecular biology-grade water and were quantified on modified Sequeira semi-selective solid medium.

**Table 1:** Accuracy assessment on pure culture of target and non-target strains related to the *Ralstonia solanacearum* species complex and other related families.

Strain	Description					93F/93R^1^	5F/5R^1^
	Alternative ID	Isolation Host	Country	Phylotype	Sequevar		
**9–1**		Bluggoe banana	Grenada	IIA	6	++-/++-	—-/—-
**A3909**		*Heliconia rostrata*	USA	IIA	6	+++/+++	—-/—-
**GMI8044**	BA7	Cavendish banana	Grenada	IIA	6	+++/+++	—-/—-
**GRE T11L1**		*Solanum lycopersicum*	Grenada	IIA	6	+++/-++	—-/—-
**GUY B06E2**		Cavendish banana	French Guiana	IIA	6	-++/-+-	—-/—-
**UQRS457**		*Heliconia rostrata*	Hawaii	IIA	6	+++/++-	—-/—-
**UW588**		Cavendish banana	Guatemala	IIA	6	+-+/+—	—-/—-
**B26**		*Musa* spp.	Brazil	IIA	24	+—/++-	—-/—-
**B34**		*Musa* spp.	Brazil	IIA	24	+++/+++	—-/—-
**B43**		*Musa* spp.	Brazil	IIA	24	+++/+++	—-/—-
**B50**		*Musa* spp.	Brazil	IIA	24	+++/+++	—-/—-
**B91**		*Musa* spp.	Brazil	IIA	24	+++/+++	—-/—-
**IBSBF1900**		*Musa* spp.	Brazil	IIA	24	+++/+++	—-/—-
**CFBP1183**	UQRS35, JS793; 07–027	*Heliconia rostrata*	Costa Rica	IIB	3	+++/+++	—-/—-
**CFBP1416**	K138; JS746; JS748	Plantain banana	Costa Rica	IIB	3	+++/+++	—-/—-
**CIP414**	Bug14	*Musa* spp.	Philippines	IIB	3	+++/+-+	—-/—-
**CIP417**	Bug2	*Musa* spp.	Philippines	IIB	3	+-+/+-+	—-/—-
**JT644**	UW9; S147	*Heliconia rostrata*	Costa Rica	IIB	3	+++/+++	—-/—-
**UW11**	S167; K207	*Heliconia rostrata*	Costa Rica	IIB	3	+-+/-++	—-/—-
**UW166**	UQRS18, CIP27	Plantain banana	Costa Rica	IIB	3	+++/+++	—-/—-
**UW2**	UQRS17, R377, CIP2	*Musa* spp.	Costa Rica	IIB	3	+++/+++	—-/—-
**UW28**		*Solanum tuberosum*	Cyprus	IIB	3	+++/+++	—-/—-
**CFBP1415**	JS740; K82	*Solanum tuberosum*	Colombia	IIB	4	+++/+++	—-/—-
**CFBP1418**	JS790	*Heliconia rostrata*	Costa Rica	IIB	4	++-/+++	—-/—-
**LNPV31.10**	2006 539	*Musa* spp.	French Guiana	IIB	4	-++/-++	—-/—-
**SVG B09B1**		*Musa* spp.	Saint Vincent	IIB	4	++-/+++	—-/—-
**UW160**	GMI8138; S253	Plantain banana	Peru	IIB	4	+++/+-+	—-/—-
**UW163**	S256; GMI8235	Plantain banana	Peru	IIB	4	+++/-++	—-/—-
**UW170**		*Heliconia rostrata*	Colombia	IIB	4	+++/+++	—-/—-
**UW179**	R368; CIP30; K254	Plantain banana	Colombia	IIB	4	+++/+++	—-/—-
**ANT80**	CIR02–080	*Anthurium andreanum*	Martinique	IIB	4NPB	+++/+-+	+++/+++
**CFBP6780**	02–143–1; 8283	*Solanum lycopersicum*	Martinique	IIB	4NPB	+++/+++	+++/+++
**CFBP6783**	ANT75; CIR02–075	*Heliconia caribea*	Martinique	IIB	4NPB	+++/+++	+++/+++
**CFBP6797**	PV8; SPV02–30308; 8280	*Solanum americanum*	Martinique	IIB	4NPB	+++/+++	+++/+++
**CFBP7014**	06–024; 8291	*Anthurium andreanum*	Trinidad	IIB	4NPB	+++/+-+	+++/+-+
**IBSBF1454**		*Cucurbita pepo*	Brazil	IIB	4NPB	+-+/+-+	+++/+++
**IBSBF1503**		*Cucumis sativus*	Brazil	IIB	4NPB	+++/+++	-++/+++
**LNPV24.25**	2001 868	*Solanum lycopersicum*	France	IIB	4NPB	++-/+—	+++/+++
**LNPV30.75**	2006 0261	*Capsicum annuum*	French Guiana	IIB	4NPB	+-+/+—	+++/+++
**PV1**	SPV02–60196	*Solanum melongena*	Martinique	IIB	4NPB	+-+/-++	+++/+++
**R288**	CFBP6442; UW373	*Morus alba*	China	I	12	—-/—-	—-/—-
**CFBP7058**	CMR134	*Vaccinium membranaceum*	Cameroon	I	13	—-/—-	—-/—-
**PSS4**		*Solanum lycopersicum*	Taiwan	I	15	—-/—-	—-/—-
**ACH92**	CFBP6425	*Zingiber officinale*	Australia	I	16	—-/—-	—-/—-
**IBSBF1882**		*Begonia semperflorens*	Brazil	I	17	—-/—-	—-/—-
**GMI1000**	JS753	*Solanum lycopersicum*	French Guiana	I	18	—-/—-	—-/—-
**JT519**		*Pelargonium asperum*	Reunion	I	31	—-/—-	—-/—-
**CIP365**	WP144	*Solanum tuberosum*	Philippines	I	45	—-/—-	—-/—-
**MAD-017**		*Capsicum annuum*	Madagascar	I	46	—-/—-	—-/—-
**GMI8254**	TO1	*Solanum lycopersicum*	Indonesia	I	47	—-/—-	—-/—-
**K60**	CFBP2047; LMG2299T	*Solanum lycopersicum*	USA	IIA	7	—-/—-	—-/—-
**RF32**		*Solanum lycopersicum*	Trinidad	IIA	7	—-/—-	—-/—-
**CIV30**		*Solanum lycopersicum*	IvoryCoast	IIA	35	—-/—-	—-/—-
**CFBP2957**	MT5	*Solanum lycopersicum*	Martinique	IIA	36	—-/—-	—-/—-
**CFBP2958**	GT4	*Solanum lycopersicum*	Guadeloupe	IIA	39	—-/—-	—-/—-
**JQ1143**		*Solanum tuberosum*	Reunion	IIA	39	—-/—-	—-/—-
**CIP239**	UW469	*Solanum tuberosum*	Brazil	IIA	40	—-/—-	—-/—-
**AP31H**		*Solanum tuberosum*	Uruguay	IIB	1	—-/—-	—-/—-
**CFBP1417**		*Solanum tuberosum*	Australia	IIB	1	—-/—-	—-/—-
**CFBP3858**	JS907; PD2763	*Solanum tuberosum*	Netherlands	IIB	1	—-/—-	—-/—-
**IPO1609**		*Solanum tuberosum*	Netherlands	IIB	1	—-/—-	—-/—-
**JT516**		*Solanum tuberosum*	Reunion	IIB	1	—-/—-	—-/—-
**LNPV19.66**		*Solanum tuberosum*	France	IIB	1	—-/—-	—-/—-
**UW551**		*Pelargonium asperum*	Kenya	IIB	1	—-/—-	—-/—-
**CFBP3879**	PD1958; CFBP1414	*Solanum tuberosum*	Colombia	IIB	2	—-/—-	—-/—-
**DGBBC1138**		*Solanum tuberosum*	Guinea	III	43	—-/—-	—-/—-
**CMR33**		*Solanum lycopersicum*	Cameroon	III	20	—-/—-	—-/—-
**CFBP6942**	CMR32	*Vaccinium membranaceum*	Cameroon	III	29	—-/—-	—-/—-
**CMR15**	CFBP6941	*Solanum lycopersicum*	Cameroon	III	29	—-/—-	—-/—-
**CMR20**		*Solanum lycopersicum*	Cameroon	III	29	—-/—-	—-/—-
**CFBP3059**	JS904	*Solanum melongena*	Burkina Faso	III	23	—-/—-	—-/—-
**CFBP7038**	CMR66	*Vaccinium membranaceum*	Cameroon	III	49	—-/—-	—-/—-
**JT525**		*Pelargonium asperum*	Reunion	III	19	—-/—-	—-/—-
**NCPPB1029**	B509	*Pelargonium asperum*	Reunion	III	19	—-/—-	—-/—-
**NCPPB1018**	JS950	*Solanum tuberosum*	Angola	III	21	—-/—-	—-/—-
**MAFF301558**	06–042	*Solanum tuberosum*	Japan	IV	8	—-/—-	—-/—-
**JT663**	R008	*Syzygium aromaticum*	Indonesia	IV	9	—-/—-	—-/—-
**R24**	UQRS466	*Syzygium aromaticum*	Indonesia	IV	9	—-/—-	—-/—-
**R28**		*Syzygium aromaticum*	Indonesia	IV	9	—-/—-	—-/—-
**PSI07**	CFBP7288	*Solanum lycopersicum*	Indonesia	IV	10	—-/—-	—-/—-
**R229**	NCPPB3726	*Musa* spp.	Indonesia	IV	10	—-/—-	—-/—-
**UQRS283**	T38	*Solanum lycopersicum*	Indonesia	IV	10	—-/—-	—-/—-
**UQRS627**	1712075KkB-PWR	*Musa* spp.	Indonesia	IV	10	—-/—-	—-/—-
**UQRS633**	240808RB-MND	*Musa* spp.	Indonesia	IV	10	—-/—-	—-/—-
**ACH732**	CIP357	*Solanum lycopersicum*	Australia	IV	11	—-/—-	—-/—-
**LMG5942T**	*Ralstonia pickettii*					—-/—-	—-/—-
**LMG21421**	*Ralstonia insidiosa*					—-/—-	—-/—-
**LMG6866T**	*Ralstonia mannitolytica*					—-/—-	—-/—-
**LMG1199T**	*Ralstonia eutropha*					—-/—-	—-/—-
**LMG2172T**	*Pseudomonas corrugata*					—-/—-	—-/—-
**LMG5093**	*Pseudomonas syringae* pv. *tomato*					—-/—-	—-/—-
**LMG2162T**	*Pseudomonas cichorii*					—-/—-	—-/—-
**LMG16206**	*Pseudomonas putida*					—-/—-	—-/—-
**LMG1794T**	*Pseudomonas fluorescens*					—-/—-	—-/—-
**LMG2404T**	*Pectobacterium corotovorum* subsp. *carotovorum*					—-/—-	—-/—-
**LMG2129T**	*Burkholderia andropogonis*					—-/—-	—-/—-
**LMG1222T**	*Burkholderia cepacia*					—-/—-	—-/—-
**LMG2804T**	*Erwinia chrysanthemi*					—-/—-	—-/—-
**LMG2894**	*Clavibacter michiganensis* subsp. *sepedonicus*					—-/—-	—-/—-
**LMG7333**	*Clavibacter michiganensis* subsp. *michiganensis*					—-/—-	—-/—-
**NCPPB881T**	*Xanthomonas gardneri*					—-/—-	—-/—-
**NCPPB4321T**	*Xanthomonas perforans*					—-/—-	—-/—-
**LMG911T**	*Xanthomonas vesicatoria*					—-/—-	—-/—-
**NCPPB2968T**	*Xanthomonas euvesicatoria*					—-/—-	—-/—-
**CFBP5827**	*Xanthomonas campestris* pv. *raphani*					—-/—-	—-/—-
**CFBP7171**	*Xanthomonas vasicola* pv. *musacearum*					—-/—-	—-/—-
**BBTV_NvllCal**	Banana bunchy top virus^3^					—-/—-	—-/—-
**CMV011**	Cucumber mosaic virus^3^					—-/—-	—-/—-
**BSV-001**	Banana streak virus^3^					—-/—-	—-/—-
**BBrMV008**	Banana bract mosaic virus^3^					—-/—-	—-/—-
**BanMMV001**	Banana mild mosaic virus^3^					—-/—-	—-/—-
**Inclusivity** ^**2**^						93% *[0*.*86–0*.*97]*	100% *[0*.*88–1]*
**Exclusivity** ^**2**^						100% *[0*.*98–1]*	100% *[0*.*99–1]*
**Accuracy** ^**2**^						97%[0.95–0.99]	100%[0.99–1]

^1^ ‘+’: positive result; ‘-’: negative result; two PCR reactions per sample were repeated three times (separated by a slash)

^2^ calculated according to the ratio of agreements relatively to the total repetitions performed within the experiment (agreements and disagreements); numbers in brackets indicates the binomial exact proportion confidence interval

^3^ viruses were tested on plant extracts. Codification for international collection are as follow: IBSBF: Biological Institute Culture Collection of Phytopathogenic Bacteria (Brazil); CFBP: Collection Française de Bactéries Phytopathogènes (French Collection of Phytopathogenic Bacteria) (France); BCCM/LMG: Belgian Co-ordinated Collections of Micro-organisms/ Laboratory of Microbiology of Ghent (Belgium); NCPPB: National Collection of Plant Pathogenic Bacteria (UK); MAFF: Ministry of Agriculture, Forestry and Fisheries Genebank (Japan).

The phylotype multiplex PCR (Pmx-PCR) developed by Fegan and Prior [6] was used to confirm that the strains belonged to the Rssc and to determine their phylotypes. Partial *egl* sequencing was performed to determine the sequevars [22]. The Moko lineage strains were typed using the Moko multiplex PCR (Mmx-PCR) designed to identify Moko strains in the historic phylotypes IIA-6, IIB-3, IIB-4, IIB-4NPB [19], and IIA-24 [20].

Viruses were obtained from lyophilized reference samples in the Plant Health Laboratory (Anses-Reunion Island).

No DNA extraction was required during the workflow.

### Duplex PCR assay development

Marker selection was performed by conducting genome comparisons using the Gene Phyloprofile Exploration tool (default parameters) of the MicroScope platform [23] (Genoscope, Evry, France) of both the Moko and IIB-4NPB strains versus the non-target strains from the Rssc and non-Rssc databases (available online: https://www.genoscope.cns.fr/agc//microscope/about/microscopeprojects.php?P_id=67). The fully sequenced and annotated genomes of Moko-related strains included the following: Grenada 9–1 (IIA-6), UW181 (IIA-6), B50 (IIA-24), IBSBF1900 (IIA-24), CFBP1416 (IIB-3), CIP417 (IIB-3), Molk2 (IIB-3), UW163 (IIB-4), and UW179 (IIB-4). Genomes from IIB-4NPB strains included CFBP6783, IBSBF1503, and CFBP7014. Primers were designed using Primer3 [24,25] running under Geneious v6.1.8 (Biomatters, available at http://www.geneious.com/).

The optimized 25-μL (total volume) PCR reaction mixture contained 1x PCR GoTaq Green Buffer Mix and 0.625 U of GoTaq Hot Start polymerase (Promega, Madison, WI, USA), 1.5 mM MgCl_2_, 0.2 mM dNTPs, 2 μM each of the 4 primers, and 2 μL of the analysis strain suspended in molecular biology-grade water. The PCR amplification was performed using a Veriti thermal cycler (Applied Biosystems, Carlsbad, CA, USA) with the following parameters: 5 min at 96°C, followed by 35 cycles of 94°C for 15 s, 70°C for 30 s, and 72°C for 30 s, with a final extension step for 10 min at 72°C.

### Performance assessment

#### Accuracy (analytical specificity)

The performance of the two new sets of primers to detect Moko disease-causing strains and 4NPB variant strains was fully evaluated, as required in the ISO 17025 standard [26] (general requirements for the competence of testing and calibration laboratories), by following European and Mediterranean Plant Protection Organization (EPPO) protocols [27,28], thereby ensuring the highest confidence in method development and validation. The assessment first involved the evaluation of the accuracy, referred to as “analytical specificity” in the EPPO standard [27], which relies on the qualitative detection capacity of the method and that includes two criteria, namely inclusivity and exclusivity. Inclusivity is the ability to avoid false negatives, while exclusivity is the ability to avoid false positives. The accuracy evaluation was performed using the duplex PCR assay with 111 strains (Table 1; n = 40 target strains; n = 71 non-target strains) at a concentration of 10^8^ CFU/mL in molecular biology-grade water. The inclusivity and the exclusivity were assessed for three replicates and were calculated using the ratio of agreement (true positives) relative to the total repetitions performed within the experiment (agreements and disagreements). Accuracy was calculated following the same exact methods as for the inclusivity and exclusivity (Table 1). Binomial exact proportion confidence interval was calculated for all the three parameters, namely inclusivity, exclusivity, and accuracy scores (R statistical softwarev3.1.2 [29], package “STATS” function “binom.test”). PCR was performed on pure cultured strains.

#### Detectability (analytical sensitivity) and repeatability

Second, the assessment involved the evaluation of the detectability, referred to as “analytical sensitivity” in the EPPO standard [27], and repeatability (EPPO standard), which relies on the quantitative detection capacity of target strains. Detectability refers to the smallest amount of target that can be reliably detected, and repeatability characterizes the level of agreement among replicates of a sample tested under the same conditions. The detectability and repeatability evaluation was performed using the primer sets 93F/93R and 5F/5R in simplex PCR (Table 2, n = 15, and Table 3, n = 10) and in duplex PCR (Table 4, n = 22). To simulate routine laboratory conditions, this test was performed using strains calibrated at 10^8^ CFU/mL in molecular biology-grade water that were successively diluted to 6 different concentrations, ranging from 10^6^ to 10^1^ CFU/mL, with banana pseudo-stem extracts (2.0 g of pseudo-stems were ground in 5 mL of molecular biology-grade water). As above, the detectability and repeatability were assessed for three replicates and were calculated according to the ratio of agreement (true positives) to the total repetitions performed within the experiment (agreements and disagreements). PCR was performed on spiked plant extracts.

**Table 2:** Detectability and repeatability assessment in artificially contaminated plant extracts for primers 93F/93R specific to Moko disease-causing strains and IIB-4NPB strains.

Strain	Phylotype-Sequevar	Concentration (CFU/mL)^1^					
		10^6^	10^5^	10^4^	10^3^	10^2^	10^1^
**GMI8044**	IIA-6	+++/+++	+++/+++	+++/—+	—-/—-	—-/—-	—-/—-
**9–1**	IIA-6	+++/+++	+++/+++	+++/+-+	—+/—+	—-/—-	—-/—-
**A3909**	IIA-6	+++/+++	+++/+++	—-/—-	—-/—-	—-/—-	—-/—-
**IBSBF1900**	IIA-24	+++/+++	+++/+++	+++/+-+	—+/—+	—-/—-	—-/—-
**B50**	IIA-24	+++/+++	+++/+++	+-+/+-+	—-/—+	—-/—-	—-/—-
**B26**	IIA-24	+++/+++	+++/+++	++-/++-	-+-/—-	—-/—-	—-/—-
**CIP417**	IIB-3	+++/+++	+++/+++	+++/-++	—+/-++	—-/—+	—-/—-
**CFBP1416**	IIB-3	+++/+++	+++/+++	+++/-++	—+/-++	—-/—+	—-/—-
**MOLK2**	IIB-3	+++/+++	+++/+++	+-+/+++	—+/+-+	—-/—-	—-/—-
**UW160**	IIB-4	+++/+++	+++/+++	—+/-++	+—/—-	—-/—-	—-/—-
**UW163**	IIB-4	+++/+++	+++/+++	—+/-++	—-/—-	—-/—-	—-/—-
**UW179**	IIB-4	+++/+++	+++/+++	+-+/++-	—-/—-	—-/—-	—-/—-
**CFBP6780**	IIB-4NPB	+++/+++	+++/+++	+—/—-	—-/—-	—-/—-	—-/—-
**CFBP6783**	IIB-4NPB	+++/+++	+++/+++	—-/—-	—-/—-	—-/—-	—-/—-
**CFBP7014**	IIB-4NPB	+++/+++	+++/+++	—-/—-	—-/—-	—-/—-	—-/—-
**Detectability** ^**2**^		**100%**	**100%**	**67%**	**24%**	**4%**	**0%**
**Repeatability** ^**2**^		**100%**	**100%**	**80%**	**80%**	**100%**	**100%**

^1^ ‘+’: positive result; ‘-’: negative result; two PCR reactions per sample were repeated three times (separated by a slash)

^2^ calculated according to the ratio of agreements relatively to the total repetitions performed within the experiment (agreements and disagreements).

**Table 3:** Detectability and repeatability assessment in artificially contaminated plant extracts for primers 5F/5R specific to IIB-4NPB strains.

Strain	Phylotype-Sequevar	Concentration (CFU/mL)^1^					
		10^6^	10^5^	10^4^	**10** ^**3**^	**10** ^**2**^	**10** ^**1**^
**ANT80**	IIB-4NPB	+++/+++	+++/+++	+-+/+—	—-/-+-	—-/—-	—-/—-
**CFBP6780**	IIB-4NPB	+++/+++	+++/+++	+—/+—	—-/—+	—-/—-	—-/—-
**CFBP6783**	IIB-4NPB	+++/+++	+++/+++	+—/+—	—+/—+	—-/—-	—-/—-
**CFBP6797**	IIB-4NPB	+++/+++	+++/+++	+—/-++	+—/—-	—-/—-	—-/—-
**CFBP7014**	IIB-4NPB	+++/+++	+++/+++	—+/-++	—-/—-	—-/—-	—-/—-
**IBSBF1454**	IIB-4NPB	+++/+++	+++/+++	+—/—+	—-/—+	—-/—-	—-/—-
**IBSBF1503**	IIB-4NPB	+++/+++	+++/+++	+—/+—	—-/—-	—-/—-	—-/—-
**LNPV24.25**	IIB-4NPB	+++/+++	+++/+++	-+-/-+-	—+/—-	—-/—-	—-/—-
**LNPV30.75**	IIB-4NPB	+++/+++	+++/+++	—+/—+	—-/—-	—-/—-	—-/—-
**PV1**	IIB-4NPB	+++/+++	+++/+++	+—/-+-	—+/—+	—-/—-	—-/—-
**Detectability** ^**2**^		**100%**	**100%**	**53%**	**23%**	**0%**	**0%**
**Repeatability** ^**2**^		**100%**	**100%**	**90%**	**83%**	**100%**	**100%**

^1^ ‘+’: positive result; ‘-’: negative result; two PCR reactions per sample were repeated three times (separated by a slash)

^2^ calculated according to the ratio of agreements relatively to the total repetitions done within the experiment (agreements and disagreements).

**Table 4:** Detectability and repeatability assessment in artificially contaminated plant extracts of the duplex-PCR 93F/93R & 5F/5R for *Ralstonia solanacearum* Moko disease-causing strains and IIB-4NPB strains.

Strain	Phylotype-Sequevar	Concentration (CFU/mL)^1^					
		10^6^	10^5^	10^4^	10^3^	10^2^	10^1^
**GMI8044**	IIA-6	+++/+++	+++/+++	-++/-++	—-/—+	—-/—-	—-/—-
**9–1**	IIA-6	+++/+++	+++/+++	-++/—+	+—/—-	—-/—-	—-/—-
**A3909**	IIA-6	+++/+++	+++/+++	+—/+—	—-/—-	—-/—-	—-/—-
**IBSBF1900**	IIA-24	+++/+++	+++/+++	-++/-++	-+-/—-	—-/—-	—-/—-
**B50**	IIA-24	+++/+++	+++/+++	+-+/+—	+—/—-	—-/—-	—-/—-
**B26**	IIA-24	+++/+++	+++/+++	++-/++-	—-/—-	—-/—-	—-/—-
**CIP417**	IIB-3	+++/+++	+++/+++	—+/-++	—+/—+	—-/—-	—-/—-
**CFBP1416**	IIB-3	+++/+++	+++/+++	-+-/-+-	—-/—-	—-/—-	—-/—-
**MOLK2**	IIB-3	+++/+++	+++/+++	+—/+—	+—/—-	—-/—-	—-/—-
**UW160**	IIB-4	+++/+++	+++/+++	—-/—-	—-/—-	—-/—-	—-/—-
**UW163**	IIB-4	+++/+++	+++/+++	+—/+—	—-/—-	—-/—-	—-/—-
**UW179**	IIB-4	+++/+++	+++/+++	-++/-++	—-/—-	—-/—-	—-/—-
**ANT80**	IIB-4NPB	+++/+++	+++/+++	-+-/-+-	-+-/-+-	—-/—-	—-/—-
**CFBP6780**	IIB-4NPB	+++/+++	+++/+++	—+/—+	—-/—-	—-/—-	—-/—-
**CFBP6783**	IIB-4NPB	+++/+++	+++/+++	+—/+—	-+-/-+-	—-/—-	—-/—-
**CFBP6797**	IIB-4NPB	+++/+++	+++/+++	—-/—-	—-/—-	—-/—-	—-/—-
**CFBP7014**	IIB-4NPB	+++/+++	+++/+++	++-/++-	++-/++-	—-/—-	—-/—-
**IBSBF1454**	IIB-4NPB	+++/+++	+++/+++	—-/—-	—-/—-	—-/—-	—-/—-
**IBSBF1503**	IIB-4NPB	+++/+++	+++/+++	+—/+—	+—/+—	—-/—-	—-/—-
**LNPV24.25**	IIB-4NPB	+++/+++	+++/+++	—+/—+	—+/—+	—-/—-	—-/—-
**LNPV30.75**	IIB-4NPB	+++/+++	+++/+++	-++/-++	—-/—-	—-/—-	—-/—-
**PV1**	IIB-4NPB	+++/+++	+++/+++	—-/—-	—-/—-	—-/—-	—-/—-
**Detectability** ^**2**^		**100%**	**100%**	**41%**	**18%**	**0%**	**0%**
**Repeatability** ^**2**^		**100%**	**100%**	**95%**	**92%**	**100%**	**100%**

^1^ ‘+’: positive result; ‘-’: negative result; two PCR reactions per sample were repeated three times (separated by a slash)

^2^ calculated according to the ratio of agreements relatively to the total repetitions performed within the experiment (agreements and disagreements).

#### Control

Several controls were used throughout the evaluation of the protocol, including a negative process control (non-target strain), a positive process control (strains UW28 (IIB-4) and CFPB7014 (IIB-4NPB)), and a negative PCR control (water). The banana plants used (Musa *acuminata* cv. 911) (AAA-Cavendish) were obtained *in vitro* from Vitropic (Saint-Mathieu-de-Tréviers, France) and grown in nurseries until the 5 true leaf stage. The plants were previously typed as *R*. *solanacearum*-free; no symptoms of any disease were observed.

#### Selectivity

Several healthy banana cultivars obtained from Vitropic were tested to assess any cross-reactions; they included Williams (AAA-Cavendish), Flhorban 925 (AAA-Cavendish synthetic hybrid), 902 (AAA-Cavendish), Brimbo 1 (AAB-plantain), Corne (AAB-plantain), Creole French (AAB-French Horn), and Big Ebanga (AAB-plantain).

### Full-length protocol test

**Fig 1:**
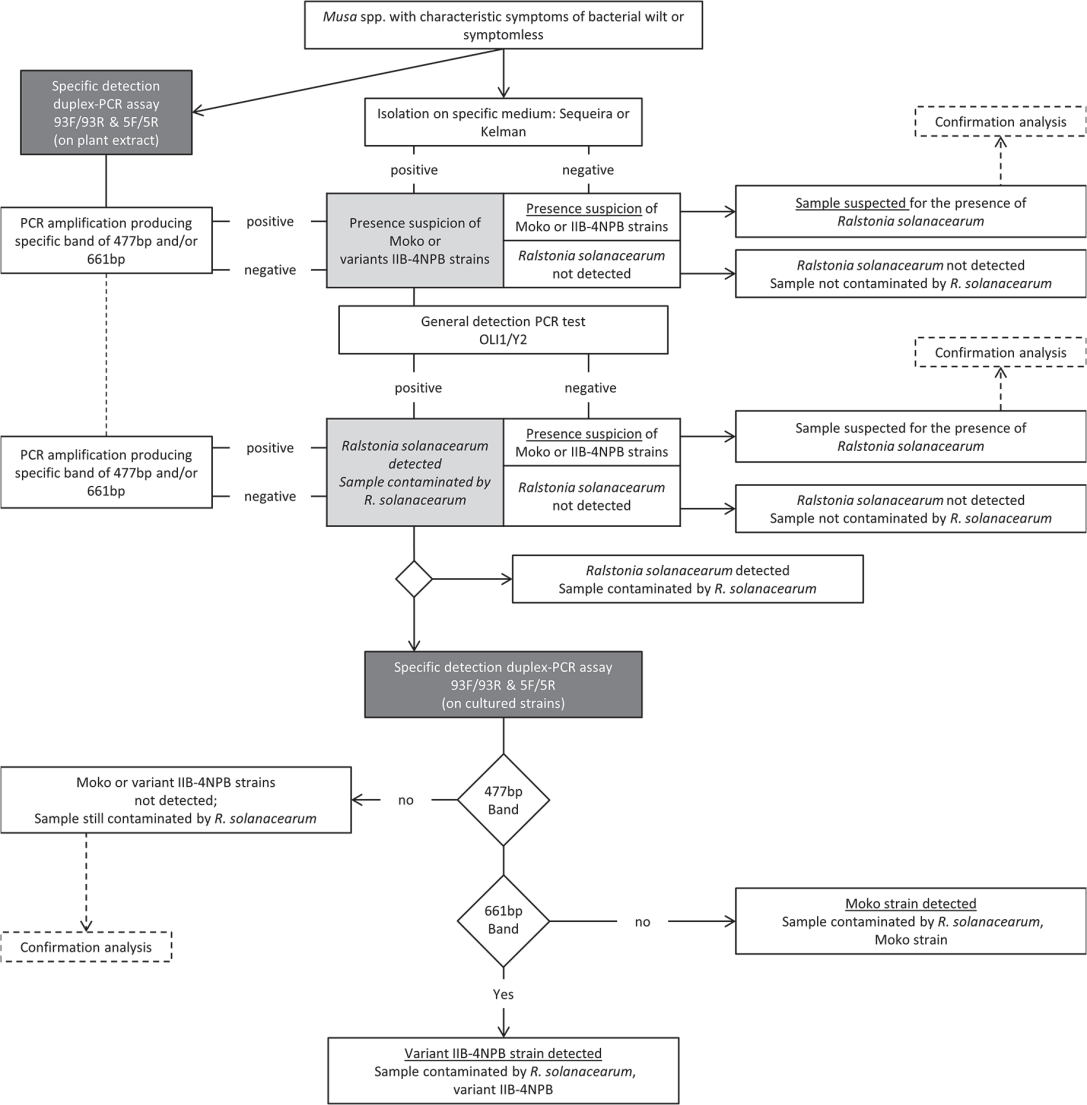
Detection scheme of *Ralstonia solanacearum* phylotype II strains in *Musa* spp.: Moko and variant IIB-4NPB.

In addition, a full-length protocol assessment (Fig. 1) was conducted on a set of 15 strains (Table 5) and was repeated twice. Each targeted group was assessed for detection by following the steps of the proposed scheme (Fig. 1). A plant extract solution, composed of 2.0 g of pseudo-stems ground in 5 mL of molecular biology grade water, was used to adjust the concentration of the Rs strains to 10^5^ CFU/mL. This spiked plant extract (50 μL) was plated on solid modified Sequeira medium for colony isolation. Typical colonies (pinkish color in the center and creamy white color on the side, smooth aspect, average elevation, and irregular border) were retrieved and characterized by PCR. We first performed PCR with the primer set Oli1/Y2 [13], then with the Mmx-PCR [19,20], and finally with the duplex PCR assay with 93F/93R and 5F/5R. PCR was conducted only on pure cultures; no DNA extraction was performed. In parallel with plating, plant extracts were also tested using the same PCR protocols.

**Table 5:** Results of the proposed full detection protocol for detection of *Ralstonia solanacearum* in artificially contaminated plant extracts and identification of isolated bacteria.

Strain	Phylotype	Sequevar	Sequeira^1^	Oli1/Y2^2^		*Musa* Mmx^2^		Duplex 93/5^2^ ^,^ ^3^	
				Plant Extracts	Pure Culture	**Plant Extracts**	**Pure Culture**	**Plant Extracts**	**Pure Culture**
**A3909**	IIA	6	+/+	++/++	++/++	++/++	++/++	++/++	++/++
**GMI8044**	IIA	6	+/+	++/++	++/++	++/++	++/++	++/++	++/++
**UW588**	IIA	6	+/+	++/++	++/++	++/++	++/++	++/++	++/++
**B43**	IIA	24	+/+	++/++	++/++	++/++	++/++	++/++	++/++
**B50**	IIA	24	+/+	++/++	++/++	++/++	++/++	++/++	++/++
**B91**	IIA	24	+/+	++/++	++/++	++/++	++/++	++/++	++/++
**Molk2**	IIB	3	+/+	++/++	++/++	++/++	++/++	++/++	++/++
**CFBP1183**	IIB	3	+/+	++/++	++/++	++/++	++/++	++/++	++/++
**UW28**	IIB	3	+/+	++/++	++/++	++/++	++/++	++/++	++/++
**UW163**	IIB	4	+/+	++/++	++/++	++/++	++/++	++/++	++/++
**UW170**	IIB	4	+/+	++/++	++/++	++/++	++/++	++/++	++/++
**UW179**	IIB	4	+/+	++/++	++/++	++/++	++/++	++/++	++/++
**CFBP6780**	IIB	4NPB	+/+	++/++	++/++	++/++	++/++	++/++	++/++
**CFBP6783**	IIB	4NPB	+/+	++/++	++/++	++/++	++/++	++/++	++/++
**IBSBF1503**	IIB	4NPB	+/+	++/++	++/++	++/++	++/++	++/++	++/++

^1^ ‘+’: positive result; ‘-’: negative result; two platings were performed on the modified Sequeira semi-selective medium per sample (separated by a slash)

^2^ ‘+’: positive result; ‘-’: negative result; two PCR reactions per sample were repeated twice (separated by a slash)

^3^ Results given by the duplex-PCR 93F/R & 5F/R were in accordance with the type of strain: Moko or IIB-4NPB.

### Newly described Moko disease-causing strains from Brazil

A final set of 18 DNA extracts from the newly described Brazilian Moko disease-causing strains [17] from sequevars IIA-41, IIA-53, and IIB-25, along with an extra set of 5 previously known strains of sequevars IIA-41 and IIB-25 (Table 6) associated with *Solanaceae* wilt but unable to cause wilt in banana plants, was assessed and repeated twice. These sets were assessed by PCR using Oli1/Y2 PCR [13], by Mmx-PCR [19,20], and by the new duplex PCR assay developed in this study.

**Table 6:** PCR comparison for the detection of *Ralstonia solanacearum* new Moko disease-causing strains from Brazil (DNA) and related *Solanaceae* strains from same sequevar (pure culture).

Strain	Isolation Host	Country	Phylotype	Sequevar	Oli1/Y2^1^	*Musa* Mmx^1^	Duplex 93F/93R & 5F/5R^1^ ^,^ ^2^
**B105**	*Musa* spp.	Brazil	IIA	41	++/++	—/—	++/++
**B54**	*Musa* spp.	Brazil	IIA	41	++/++	—/—	++/++
**B64**	*Musa* spp.	Brazil	IIA	41	++/++	—/—	++/++
**B66**	*Musa* spp.	Brazil	IIA	41	++/++	—/—	++/++
**B73**	*Musa* spp.	Brazil	IIA	41	++/++	—/—	++/++
**B74**	*Musa* spp.	Brazil	IIA	41	++/++	—/—	++/++
**B75**	*Musa* spp.	Brazil	IIA	41	++/++	—/—	++/++
**B95**	*Musa* spp.	Brazil	IIA	41	++/++	—/—	++/++
**B96**	*Musa* spp.	Brazil	IIA	41	++/++	—/—	++/++
**B106**	*Musa* spp.	Brazil	IIA	41	++/++	—/—	++/++
**BV136**	*Musa* spp.	Brazil	IIA	41	++/++	—/—	++/++
**Cotpin2**	*Musa* spp.	Brazil	IIA	53	++/++	—/—	++/++
**F2**	*Musa* spp.	Brazil	IIA	53	++/++	—/—	++/++
**F3**	*Musa* spp.	Brazil	IIA	53	++/++	—/—	++/++
**IBSBF2572**	*Musa* spp.	Brazil	IIA	53	++/++	—/—	++/++
**B4**	*Musa* spp.	Brazil	IIB	25	++/++	—/—	++/++
**B7**	*Musa* spp.	Brazil	IIB	25	++/++	—/—	++/++
**B10**	*Musa* spp.	Brazil	IIB	25	++/++	—/—	++/++
**CFBP7032**	*Solanum lycopersicum*	Cameroon	IIA	41	++/++	—/—	—/—
**06037**	Water (irrigation)	French Guiana	IIA	41	++/++	—/—	—/—
**CIP10**	*Solanum tuberosum*	Peru	IIB	25	++/++	—/—	—/—
**IBSBF2001**	*Solanum lycopersicum*	Brazil	IIB	25	++/++	—/—	—/—
**UQRS607**	*Solanum tuberosum*	Iran	IIB	25	++/++	—/—	—/—

^1^ ‘+’: positive result; ‘-’: negative result; two PCR reactions per sample were repeated twice (separated by a slash)

^2^ positive results characterized strains a as part of the Moko lineage according to the only amplification of the 477bp band.

## Results

### Duplex PCR assay development

A genomic comparison was performed in two steps due to the high phylogenetic proximity of strains from phylotype IIB-4NPB to the Moko phylotype IIB-4. The first step compared Moko strains and variant IIB-4NPB strains to all the other strains of the *Ralstonia solanacearum* species complex in the Prokaryotic Genome DataBase (PkGDB) of the MicroScope platform [23], and the second step compared variant IIB-4NPB strains to all the other strains of the *Ralstonia solanacearum* species complex in the PkGDB of the MicroScope platform. Following extensive basic local alignment search tool (BLAST) searches against the NCBI genomic database to screen specific matches, two coding sequences were selected as markers. The first coding sequence was RALUWv1_4260003 (1,038 bp; GenBank accession KM387307), a putative KfrA protein (functional assignment based on the presence of a conserved amino acid motif, structural feature or limited homology) related to DNA binding proteins and a transcriptional modulator found in the UW181 genome and shared by both the Moko and NPB strains (92% pairwise identity). For this sequence, the primers 93F (5’ CGC TGC GCG GCC GTT TCA C 3’) and 93R (5’ CGG TCG CGG CAT GGG CTT GG 3’) were designed; they produce a 477-bp amplicon. The second coding sequence was RAL70v1_1150031 (1,140 bp; GenBank accession KM387308), a chemotaxis-related protein found in the CFBP7014 genome and only shared by NPB strains (96% pairwise identity). For this sequence, the primers 5F (5’ GCG CGC GAG GCT GGT GAT GT 3’) and 5R (5’ TGG GTT CGC AGG CGG ACA GC 3’) were designed; they produce a 661-bp amplicon.

### Performance of the duplex PCR assay

#### Accuracy (analytical specificity)

Accuracy is defined by both quantitative (inclusivity) and qualitative (exclusivity) criteria, thus characterizing the method using false positive/negative analysis. A total of 111 strains were selected (Table 1) to represent the Rssc strains and closely related strains. The primer set 93F/93R demonstrated 93% inclusivity (binomial exact proportion confidence ranging from 0.86 to 0.97) for the detection of Moko disease-causing strains and variant 4NPB strains. Although some replicates produced false-negative signals, all of the target strains were detected at least once among the 3 repetitions. Exclusivity reached 100%, thus characterizing this primer set as 97% accurate (binomial exact proportion confidence ranging from 0.95 to 0.99). The primer set 5F/5R demonstrated 100% inclusivity (binomial exact proportion confidence ranging from 0.88 to 1) and 100% exclusivity (binomial exact proportion confidence ranging from 0.99 to 1), thus yielding 100% accuracy (binomial exact proportion confidence ranging from 0.99 to 1). No variation in the amplification pattern was observed with any particular group of strains. Additionally, the tested viruses associated with banana diseases did not cross-react with the duplex PCR and thus yielded no amplification signal.

All controls yielded expected results, validating the process in its entire workflow.

#### Detectability (analytical sensitivity) and repeatability

Detectability is evaluated as the detection limit level, whereas repeatability is evaluated as the degree of agreement among test repetitions. The primer sets were tested in simplex PCR and then in duplex PCR to assess any potential competition issues that could lead to a lower PCR yield. Strains spiked into banana pseudo-stem extract were used to identify any potential PCR inhibitors and to simulate routine laboratory conditions. The results showed comparable outputs between the two simplex PCR assays (Tables 2 and 3) and the duplex PCR assay (Table 4), and repeatability was high and stable across the entire experiment: 100% repeatability was observed until 10^5^ CFU/mL, and the repeatability of the duplex PCR slightly dropped to 95% and 92% for 10^4^ CFU/mL and 10^3^ CFU/mL, respectively. The detection limit was shown to be 10^5^ CFU/mL. The simplex PCR showed the same pattern, with a slight drop in repeatability to 80% for 10^3^ CFU/mL when using the 93F/93R primer set. Additionally, half of the samples (n = 11) could be detected down to 10^3^ CFU/mL with the duplex PCR assay. Finally, no primer competition was observed during the experiment, as the simplex PCR did not give a stronger confidence level for detectability or repeatability than the duplex PCR at concentrations lower than 10^5^ CFU/mL.

All controls yielded expected results, validating the process in its entire workflow.

#### Selectivity

The different tested healthy cultivars did not cross-react with the duplex-PCR, and all samples did not yield any PCR amplification across three repetitions.

### Full-length protocol test

The full-length protocol, consisting of both plating and PCR amplifications (Oli1/Y2, Mmx-PCR, and the 93F/93R and 5F/5R duplex PCR assay) was conducted on the historic Moko disease-causing strains and IIB-4NPB strains, both in pure culture and with spiked plant extracts. The results (Table 5) show that these two lineages were detected, both on plates and with the three PCR methods, with either pure cultures or spiked plant extracts, and for both repetitions.

### Newly described Moko disease-causing strains from Brazil

The genomic DNA samples from Brazilian Moko strains showed specific amplification (Table 6) in both the Oli1/Y2 assay and the duplex 93F/93R and 5F/5R PCR assay developed in this study (characterized as part of the Moko lineage), but they were not detected by Mmx-PCR. The strains that are only pathogenic to *Solanaceae*, which cluster into sequevars IIA-41 and IIB-25 and are not related to the Moko lineage, were only amplified by the generic Oli1/Y2 primer set, which types Rs strains at the species level. Neither the Mmx-PCR nor the duplex 93F/93R and 5F/5R PCR resulted in amplification.

All controls yielded expected results, validating the process in its entire workflow.

## Discussion

The diagnostic method presented in this study for detecting Moko disease-causing strains and variant IIB-4NPB strains was developed to fit with the requirements of officially accredited diagnostic laboratories. The proposed protocol was fully evaluated, as required by the ISO 17025 standard, by following EPPO protocols, thus ensuring the highest confidence in the method development and validation.

The focus of this study was on the development of a method for the detection of the Moko lineage, which is capable of causing wilt in *Musaceae* plants. This duplex PCR assay was able to detect the historical diversity of Moko strains (sequevars IIA-6, IIA-24, IIB-3, and IIB-4) and also the newly discovered Moko-related Brazilian strains that cluster into sequevars IIB-25, IIA-41, and IIA-53. The epidemiologically variant IIB-4NPB strains that cause latent infection within the vascular system of plantains were also detected in this duplex PCR assay framework. The protocol developed in this work appears suitable for research and diagnostic laboratories and showed reliable accuracy, detectability, and repeatability (Tables 1 through 6).

Currently, no specific diagnostic protocol related to Rssc strains that are pathogenic to *Musaceae* plants has been defined by the European Commission (in the European Commission Council Directive 2000/29/EC [30] on protective measures against the introduction into the community of organisms harmful to plants or plant products and against their spread within the community or in Council Directive 2006/63/EC [31]). In addition, it is known that some strains within phylotype II that cause banana and *Musaceae* wilt are also able to wilt *Solanaceae* [21] and may represent a threat to both plant families; phylotype II *Musaceae*-adapted strains might be carried by *Solanaceae* plants. Therefore, a general assay to detect Rssc strains (e.g., with primer pairs Oli1/Y2 [13] or 759/760 [32]) and a specific Brown rot detection protocol [33] for *Solanaceae* should be used along with a specific Rs banana wilt detection protocol to detect both types of strains.

The performance assessment of the duplex PCR assay was fully compliant with its use as a reference method for diagnostic laboratories. Moko and the epidemiologically variant IIB-4NPB strains were both specifically detected with confidence. Amplification of the 477-bp Moko band could rarely fail for the IIB-4NPB strains, positive detection status should be considered if only the specific 661-bp 4NPB band is amplified; additional confirmation might also be necessary. The samples analyzed using this duplex PCR assay may consist of a pure cell culture isolated on solid medium or of plant extract samples in molecular biology-grade water. The detection of Moko and IIB-4NPB strains may be performed using either symptomatic or latently infected plants, as the concentration within xylem vessels is usually greater than the 10^5^ CFU/mL threshold estimated in this study [34–36]. Moreover, the new Brazilian Moko-related strains were successfully detected with this duplex PCR assay, which was even able to distinguish Brazilian Moko-related strains from non-Moko strains of sequevars IIB-25 and IIA-41 and to detect the new sequevar IIA-53. Selectivity assessment showed that no cross-amplification occurred with different banana cultivars, whether Cavendish or plantain. In the full-length protocol using a semi-selective medium and a specific PCR approach, the detection and characterization of all strains was achieved, providing a high confidence level for the integration of this protocol into official detection schemes to complete or confirm results obtained by other diagnostic technologies.

The design of Moko-specific primers first involved a genomic comparison between Moko strains and other Rssc and non-Rssc strains to obtain an optimal gene repertoire for diagnosis. The Moko lineage clusters into seven sequevars belonging to phylotype IIA and IIB: the previously reported Moko sequevars IIA-6, IIA-24, IIB-3, and IIB-4, which have been sequenced, and the newly described sequevars IIA-41, IIA-53, and IIB-25, for which sequenced genomes are not yet available. The paraphyletic nature of the Moko lineage makes the search for specific gene repertoires difficult to manage, as other non-related Moko sequevars could be genetically closer while belonging to another lineage (e.g., Brown rot IIB-1 or IIB-4NPB strains).

Unsurprisingly, given the close phylogenetic proximity between IIB-4 Moko strains and IIB-4NPB strains, no locus was found to be specific only to Moko strains; thus, IIB-4NPB strains were added to the genomic search against other Rssc and non-Rssc genomes. This similarity is consistent with the phylogenetic position of the Moko IIB-4 strains as indistinguishable from IIB-4NPB strains. We could then assume that NPB strains share almost all of the genes of the Moko lineage. Nevertheless, the IIB-4NPB strains have a specific accessory repertoire of genes, in which a specific IIB-4NPB marker was found. A total of two coding sequences were found to be suitable for primer design. The 93F/R primer set was based on the specific gene repertoire shared by both the Moko and NPB strains, while the 5F/R primer set was based solely on the specific gene repertoire of the IIB-4NPB strains. Ecologically, these two lineages are different but share common traits, such as the ability to invade plantain banana xylem vessels.

## Conclusions

The research presented herein shows that the 93F/R and 5F/R duplex PCR assay offers reliable detection of phylotype II strains of the Rssc that can be retrieved from banana plants, from other *Musaceae*, or from ornamentals. The test relies on two fully validated primer sets (as required by the ISO 17025 standard) and can be implemented in accredited laboratories or research laboratories. This duplex PCR assay showed high accuracy (inclusivity and exclusivity), with correct detection of target strains without any cross-reaction within the Rssc, high sensitivity for detecting low-concentration samples, and high repeatability, even when banana pseudo-stems were added to the sample. We recommend performing a total of two PCR repetitions for a given sample, with the results interpreted as follows: two positive results imply specific amplification and two negative results imply no specific amplification. In the case of one positive and one negative result, we suggest that the PCR should be performed again; if the results again give one positive and one negative result, it will imply (as for two positive results) specific amplification. Avoiding the introduction and spread of quarantined organisms is of utmost concern, and diagnostic methods should be implemented as early as possible in the plant breeding process (*in vitro* plantlet production) to ensure a safe phytosanitary framework. This assay was designed to detect strains within the Rssc phylotype II that are able to cause wilt or latent infections in bananas or plantains. This method can also be easily used as an epidemiological surveillance protocol (Fig. 1) for phylotype II Rs strains on both symptomatic and asymptomatic *Musaceae* plants and on ornamentals. This assay could complement EC policies on protective measures to be deployed to prevent the introduction and spread of organisms that are harmful to plants or plant products.
